# Artificial Intelligence as a Safeguard for Clinical Scientific Integrity: A Human–AI Hybrid Model for Medical Peer Review

**DOI:** 10.3390/jcm15062215

**Published:** 2026-03-14

**Authors:** Maria Pina Dore, Elettra Merola, Giuseppe Lasaracina, Giovanni Mario Pes

**Affiliations:** 1 Dipartimento di Medicina, Chirurgia e Farmacia, University of Sassari, Viale San Pietro 43, 07100 Sassari, Italy; emerola@uniss.it (E.M.); gmpes@uniss.it (G.M.P.); 2 Department of Medicine, Baylor College of Medicine, One Baylor Plaza, Houston, TX 77030, USA

**Keywords:** peer review, artificial intelligence, large language models, reviewer bias, research integrity, publication ethics, AI-assisted peer review

## Abstract

Peer review is the cornerstone of scholarly publishing and, in medicine, the ultimate guarantor of the reliability of clinical evidence that informs guidelines, therapeutic strategies, and patient care. However, the current peer review system is increasingly strained by bias, abuse, and reviewer overload. Favoritism toward prominent authors, editorial “nepotism,” coercive citation practices, superficial evaluations, and even documented cases of idea theft from confidential manuscripts undermine the trustworthiness of the scientific literature upon which clinical decisions depend. In this paper, we argue that artificial intelligence (AI) and large language models (LLMs) offer a transformative opportunity to strengthen the integrity and efficiency of medical peer review. AI-driven tools can perform rapid consistency checks, detect statistical errors or plagiarism, and enforce compliance with ethical and methodological standards across thousands of manuscripts. Early implementations of AI-guided review platforms, plagiarism detectors, and citation-anomaly algorithms demonstrate that machine assistance can make reviews more thorough, objective, and reproducible. At the same time, we acknowledge the limitations of AI, including hallucinations, a lack of human judgment, and risks to confidentiality if misused. To address these concerns, we propose a hybrid model in which AI handles routine screening and technical tasks under strict safeguards, while human experts retain final responsibility for scientific evaluation. This human–AI partnership may represent an essential step toward improving the quality, fairness, and reliability of the clinical evidence base.

## 1. Introduction

Traditional peer review faces structural and operational challenges that may erode trust in scientific communication. In the medical field, this problem acquires an additional dimension: clinical decisions, practice guidelines, and therapeutic strategies rely entirely on the reliability of published evidence. When peer review fails, the consequences are not merely editorial or academic but may indirectly affect patient safety and the quality of care [1,2,3,4,5,6,7,8,9,10]. Decades of evidence demonstrate systemic bias and favoritism: famous authors consistently receive higher acceptance rates and better reviews [11], and “nepotistic” journals are documented in which editors rapidly publish their own work [12]. Reviewers may even harass authors or insist on citing their own papers unnecessarily, a practice the Council of Science Editors has flagged as unethical [13]. Such behaviors may contribute to perceptions of bias and reduced confidence in the integrity of the review system.

Meta-research studies suggest that certain forms of editorial bias, including disproportionate publication rates among editorial board members and institutional clustering, occur at measurable frequencies across subsets of journals, although the magnitude varies substantially by discipline and methodology [14,15]. Importantly, while documented cases of idea theft and coercive citation exist, available evidence does not suggest that such misconduct characterizes the majority of peer-review interactions [16]. Distinguishing systemic structural pressures from isolated breaches of integrity is essential for balanced assessment.

At the same time, journals are drowning in submissions. Under intense “publish-or-perish” pressure, reviewers are often overloaded and decline invitations [13]. Editors report that it can take twice as many invitations to secure reviewers as in the past, and those who agree often submit late or rushed reports [14]. One survey found that 42% of researchers now decline review requests due to workload, and just 10% of reviewers account for about 50% of all reviews [15]. Inexperienced early-career researchers are increasingly enlisted to fill the gap, raising concerns about consistency and expertise in evaluations [14].

In documented cases, reviewers have violated the fundamental trust that underlies the peer review process. Some scholars warn that if anonymity is abused (for instance, through idea theft), “researchers will lose trust in peer review” altogether. Meanwhile, unethical tactics such as coercive citation (pressuring authors to add spurious references to boost the reviewer’s preferred work) have become so common that publication ethics boards explicitly label them as malpractice [17,18]. Taken together, these flaws demand a rethinking of peer review. A recent analysis concludes that the surge in manuscripts and the shortage of reviewers mean we “need a different approach,” specifically one that integrates new technologies to support the process [19].

## 2. AI Capabilities for Improving Peer Review

Large language models (LLMs) and AI-based tools offer a potential complementary approach to supporting and strengthening peer review processes. For conceptual clarity, it is important to distinguish between (a) generative LLMs, which produce narrative summaries or structured drafts, and (b) specialized analytical or rule-based systems (e.g., statistical consistency checkers, plagiarism detectors, and citation-network algorithms). LLMs are particularly useful for summarization, checklist drafting, and linguistic analysis, but are vulnerable to hallucinations and fabricated references. By contrast, rule-based or statistical tools are more reliable for narrow detection tasks (e.g., identifying duplicated text, arithmetic inconsistencies, or anomalous citation clustering), though they lack contextual understanding.

Appropriate deployment in medical evidence review, therefore, depends on matching tool class to task complexity and risk profile.

AI can automate consistency checks and other routine analyses, allowing human experts to focus on more profound insights. For instance, AI-based screening tools can instantly flag statistical or logical errors in a manuscript; a recent report noted that an LLM could have spotted a critical mathematical mistake in minutes that human reviewers missed [20].

Specialized automated checkers (e.g., Statcheck or Paperpal) are already catching calculation errors and methodological flaws that might slip past tired reviewers. Likewise, issues such as plagiarism and citation manipulation are increasingly detectable by AI. Recent work observes that AI systems can now flag missing or vague ethics statements, undisclosed conflicts of interest, and suspicious citation patterns by analyzing text and metadata [21]. Tools such as Turnitin’s iThenticate plagiarism scanner and algorithms like the CIDRE (Citation Donors and Recipients) algorithm can uncover unusual citation cartels or outright copy-paste plagiarism in submissions. AI systems can systematically and at scale apply journal policies, potentially increasing procedural consistency across submissions.

### Emerging AI Tools and Platforms in Peer Review

Prototypes and platforms that harness AI in peer review are already emerging. For example, ReviewFlow is an AI-driven system designed to guide novice reviewers through a structured workflow. In trials, researchers using ReviewFlow produced “significantly more structured and more comprehensive reviews” than those without AI guidance, identifying manuscript weaknesses more thoroughly [22]. Similarly, the OpenReviewer project envisions using GPT-based models to draft complete peer review reports at scale. Its authors report that LLMs can deliver “consistently high-quality reviews almost instantly,” rapidly pinpointing a paper’s key strengths and flaws. However, human editors still need to refine and finalize the AI-generated drafts [23]. Even general AI research assistants, such as Elicit, are being utilized to accelerate literature assessment, allowing reviewers to extract relevant data and citations from hundreds of references in just minutes.

Major publishers have also begun encouraging innovative uses of AI in the editorial process. For instance, Sage Publications has noted that editors might use ChatGPT (5.2 version) to identify potential reviewers in a given subject area (while, of course, verifying those individuals’ credentials) [24]. JMIR Publications explicitly advocates transparency over prohibition, instructing peer reviewers to disclose any AI assistance they use rather than banning AI outright [25]. Importantly, these AI tools can also scale effortlessly. An AI can process thousands of manuscripts checking for plagiarism, statistical soundness, or biases far more consistently and quickly than an overburdened human staff or volunteer could manage.

## 3. A Hybrid Model for Human–AI Peer Review

Although this perspective does not propose a validated implementation protocol, we outline a structured conceptual workflow to clarify the role distribution and decision gates within a human AI hybrid model (Table 1), which can augment, not replace, human peer review with AI.

In this model, AI systems would handle tasks they are best at, such as initial triage and technical checks, while humans would make the final scientific judgment. For example, LLMs could pre-screen incoming submissions for basic quality and scope, identify common statistical errors or missing ethics statements, and even create a preliminary checklist-style report on the manuscript. Human experts would then review and correct the AI’s output, using their expertise to assess the study’s significance, novelty, and nuanced interpretation.

Within this framework, AI use would remain supportive rather than mandatory. Journals may adopt AI-assisted screening selectively based on editorial policy and resource availability. Crucially, AI output must be validated by designated editorial or statistical reviewers before influencing publication decisions.

In this way, routine and technical tasks could be delegated to AI systems, allowing human reviewers to focus on higher-level scientific interpretation and contextual evaluation [26].

Journal editorial boards may also utilize AI to streamline specific workflows. For example, an editor could employ an algorithm to match manuscripts with appropriate reviewers or to generate a concise summary of a paper’s key points. Still, the ultimate assignment of reviewers and the final evaluation of the research would remain in human hands. As one peer-review workshop envisioned, let “machines do screening, analysing citations and identifying peer groups… and humans… decipher whether the article adds to the scholarly record.” [27].

AI systems cannot bear legal or ethical responsibility. Ultimate accountability for publication decisions must remain with journal editors and publishers. If an AI-assisted triage process fails to detect a statistical flaw that later influences clinical guidance, responsibility rests with the human editorial structure that adopted the system.

Responsibility for validation rests with human editorial leadership. AI-generated flags or summaries should be treated as advisory signals requiring explicit confirmation. No manuscript decision should rely exclusively on automated assessment.

Accordingly, AI should be framed as a decision-support infrastructure rather than a decision-making authority.

### 3.1. Task-Oriented Functions of AI in Medical Peer Review

There is growing recognition that the current peer-review model faces sustainability challenges. Journals like eLife have begun “publishing first, then reviewing,” effectively breaking the old gatekeeper model of peer review [28]. The broader lesson from such experiments is that the evaluation of a manuscript should primarily reflect its methodological rigor, relevance, and contribution to the field rather than journal-specific dynamics.

Rather than structuring the discussion around specific platforms, it is conceptually clearer to organize AI applications by the functional tasks they support in the peer-review process. Different system classes address distinct components of the editorial workflow.

*Technical integrity screening.* A first category includes systems designed to detect objective irregularities, such as plagiarism, duplicated text, arithmetic inconsistencies, missing ethics statements, undeclared conflicts of interest, or anomalous citation patterns. These tools typically rely on rule-based algorithms, statistical cross-checking, or metadata analysis rather than generative modeling. Their strength lies in reproducibility and narrow-task reliability; their limitation is the inability to assess contextual scientific merit.

*Statistical and methodological verification.* A second functional domain involves automated cross-validation of reported p-values, confidence intervals, sample sizes, or internal numerical coherence. Such systems act as analytical auditors, capable of identifying discrepancies that may escape the attention of fatigued human reviewers. However, they may generate false positives in complex or unconventional study designs.

*Linguistic structuring and reviewer assistance.* Generative LLMs primarily operate in this domain. In secure environments, they may assist reviewers by summarizing manuscripts, generating structured checklists, highlighting sections requiring scrutiny, or drafting preliminary review outlines. Because LLMs produce probabilistic text rather than deterministic verification, their outputs remain advisory and require human validation.

*Citation network and pattern analysis.* Algorithms can also analyze citation networks at scale to identify unusually dense reciprocal citation clusters or potential cartel behavior. These systems are particularly suited to detecting systemic anomalies rather than evaluating individual scientific arguments.

*Editorial workflow optimization.* Finally, AI may support logistical functions, such as reviewer matching, through topic modeling or by synthesizing recurring themes across review reports. These applications are infrastructural and do not substitute for editorial judgment.

Organizing AI capabilities by task rather than by platform underscores a central premise of this Perspective: no single tool replaces human peer review. Instead, distinct AI capability classes may support discrete components of the workflow, provided that their limitations are explicitly recognized and responsibility remains human-centered.

Moreover, given the sensitivity of clinical research, AI integration requires clearly defined governance controls that may include all of the following:

- Use of secure, journal-approved systems with explicit data retention limits;
- Preference for on-premises or contractually governed vendor-hosted environments compliant with data protection regulations;
- Prohibition of uploading confidential manuscripts into public, non-approved chatbots;
- Comprehensive logging and audit trails documenting AI interactions;
- Role-based access controls for editors, reviewers, and technical staff;
- Routine red-teaming and adversarial testing to detect system vulnerabilities;
- Prompt governance policies defining permissible uses of AI in review;
- Mandatory disclosure statements specifying whether AI was used and for what function (e.g., summarization, statistical screening, language refinement). Such controls are essential to preserve confidentiality and the editorial duty of care.

From a regulatory perspective, AI-assisted manuscript processing must comply with applicable data protection frameworks, including the GDPR and equivalent standards in other jurisdictions. Manuscripts frequently contain unpublished clinical data, and the processing of such data by AI systems may constitute data handling subject to regulatory scrutiny.

Liability considerations also warrant attention. In cases of AI-assisted erroneous rejection or acceptance, particularly where downstream clinical impact may occur, the duty of care remains with the journal and publisher. AI systems cannot be held legally accountable.

Transparency standards should therefore require explicit disclosure of AI assistance within peer-review reports, editorial workflows, and author guidelines, specifying the nature and scope of AI involvement.

### 3.2. Evaluating Impact: Metrics for Benefit and Risk

While this article does not present an empirical validation study, any responsible integration of AI into peer review should ultimately be accompanied by systematic evaluation. The value of AI assistance cannot be assumed; it must be demonstrated through transparent and measurable indicators. At a minimum, journals adopting AI-supported workflows should assess whether these tools meaningfully improve the detection of statistical inconsistencies, reporting omissions, or methodological errors, ideally examining sensitivity and specificity relative to traditional review alone. It would also be important to explore whether AI support/enhances inter-reviewer agreement, for example, by reducing unexplained variability in recommendations across similar submissions.

Operational outcomes deserve equal attention. A reduction in time-to-decision may be beneficial, but only if it occurs without deterioration in review depth, correction rates, or post-publication amendments. Furthermore, because peer review has long been affected by structural bias, any AI-assisted model should be evaluated for its effects on equity. This includes examining whether algorithmic support mitigates or inadvertently reinforces disparities related to author nationality, gender, language background, or institutional prestige.

Finally, evaluation must extend beyond benefits to potential harms. AI systems may create a false sense of reassurance when outputs appear authoritative but contain subtle errors. Over-reliance on automated summaries could encourage superficial human oversight, a phenomenon often described as automation bias. There is also a risk that biases embedded in training data or detection algorithms could systematically amplify existing inequities rather than reduce them.

For these reasons, AI integration in medical peer review should be accompanied by ongoing monitoring, predefined quality indicators, and a willingness to recalibrate or withdraw tools if unintended consequences emerge.

## 4. Limitations and Ethical Concerns of AI in Peer Review

Scholars have appropriately emphasized that AI does not represent a comprehensive solution to the challenges of peer review. All current LLMs have well-known tendencies to “hallucinate” (generate false or nonsensical information) or to reflect biases present in their training data. Evidence suggests that contemporary generative models are not consistently objective or factually reliable and may produce fabricated references or content [29,30]. In fact, JMIR reported that ChatGPT produced a convincingly fraudulent scholarly manuscript (replete with bogus citations) in about an hour, a deceptive creation that only human reviewers eventually caught [30]. These examples underscore that AI can introduce new errors even as it identifies and corrects others. AI may also reshape the dynamics of adversarial behavior in scholarly publishing. Automated paper mills, AI-generated review farms, strategically optimized citation cartels, and synthetic manuscripts designed to evade detectors represent emerging risks. Additionally, excessive trust in AI-generated assessments could foster “automation bias,” where reviewers defer too readily to algorithmic outputs.

Moreover, AI lacks the accuracy of human intuition and moral judgment. Participants in the ReviewFlow trials, for example, “expressed concerns about using AI as part of the scientific review process,” noting that no algorithm really understands what constitutes a study’s novelty or significance in the broader context [22]. There are also clear ethical and privacy risks. Many AI services (especially free online chatbots) log or store the text that users input; thus, uploading a confidential manuscript to such a tool could inadvertently leak sensitive data. Indeed, JMIR’s policy now specifically prohibits reviewers from feeding manuscripts into unapproved AI chatbots, citing these confidentiality concerns. Finally, incorporating AI into peer review raises unresolved questions about accountability and credit. If an AI system influences a review decision or report, it is unclear how to assign responsibility for that judgment. These challenges suggest that any use of AI in peer review must be carefully overseen and guided by clear guidelines.

Empirical evaluations of AI systems demonstrate considerable variability in performance across tasks. Plagiarism detection tools, for example, often achieve high sensitivity but may generate false positives when legitimate reuse of text occurs (e.g., methodological descriptions). Conversely, statistical anomaly detection tools may fail to identify complex modeling errors that are not captured by rule-based checks.

Generative LLMs, while capable of producing coherent summaries, exhibit well-documented limitations in formal statistical reasoning and may misinterpret numerical relationships in complex analyses. Performance degradation has been observed when tasks require multi-step quantitative inference rather than linguistic pattern recognition.

Reviewer-matching algorithms based on topic modeling may also inadvertently reproduce existing structural biases if trained on historically skewed publication networks.

These limitations underscore that AI performance is task-dependent and context-sensitive rather than uniformly reliable.

## 5. Conclusions

In conclusion, current pressure on peer review suggests that the traditional system is experiencing increasing strain, particularly in medical publishing, where the reliability of peer review directly influences the quality of clinical evidence. AI and LLMs now offer powerful tools to check for consistency, detect fraud, and generate structured feedback at a scale beyond what humans alone can achieve. As Bauchner and Rivara put it, “We believe AI should be used to assist in triaging manuscripts” to handle today’s deluge of submissions [19]. A hybrid AI–human model, in which machines rapidly identify technical issues and humans evaluate their scientific and clinical significance, may represent a constructive strategy for reinforcing the integrity and reliability of the clinical evidence base. By adopting AI with transparency and strong oversight, journals can improve the fairness, rigor, and reliability of peer review while protecting the quality of the medical knowledge that ultimately informs patient care.

## Figures and Tables

**Table 1:** A structured workflow for operationalizing the hybrid model.

Stage	Process	Comments
Stage 1	AI-Assisted Technical Triage	AI systems perform standardized screening for plagiarism, statistical inconsistencies, compliance with reporting guidelines (e.g., CONSORT adherence), reference anomalies, undeclared conflicts, and formatting completeness. Outputs are flagged rather than determinative. No rejection decisions are automated at this stage.
Decision Gate 1	Human Editorial Oversight	Editors review AI-generated flags and determine whether the manuscript proceeds to peer review, requires technical revision, or warrants desk rejection based on journal scope, not solely on AI outputs.
Stage 2	AI-Augmented Reviewer Support	Reviewers may optionally use secure AI tools to generate structured checklists, summarize methodological components, or verify internal consistency. AI suggestions remain advisory.
Decision Gate 2	Human Scientific Evaluation	Human reviewers retain sole authority to evaluate novelty, clinical relevance, methodological rigor, and interpretative validity. AI outputs cannot substitute for scientific judgment
Stage 3	Editorial Synthesis	Editors integrate human reviews and may use AI tools to synthesize themes or detect inconsistencies across reports, but final publication decisions remain fully human-driven.

## Data Availability

No new data were created or analyzed in this study.

## Abbreviations

The following abbreviations are used in this manuscript:

AI	Artificial Intelligence
LLM	Large Language Model
GPT	Generative Pre-trained Transformer
JMIR	Journal of Medical Internet Research
CIDRE	Citation Donors and Recipients algorithm

## References

[1] Candal-Pedreira C., Guerra-Tort C., Ruano-Ravina A., Freijedo-Farinas F., Rey-Brandariz J., Ross J.S., Perez-Rios M. (2024). Retracted papers originating from paper mills: A cross-sectional analysis of references and citations. J. Clin. Epidemiol..

[2] Fang F.C., Steen R.G., Casadevall A. (2012). Misconduct accounts for the majority of retracted scientific publications. Proc. Natl. Acad. Sci. USA.

[3] Kataoka Y., Banno M., Tsujimoto Y., Ariie T., Taito S., Suzuki T., Oide S., Furukawa T.A. (2022). Retracted randomized controlled trials were cited and not corrected in systematic reviews and clinical practice guidelines. J. Clin. Epidemiol..

[4] Hwang S.Y., Yon D.K., Lee S.W., Kim M.S., Kim J.Y., Smith L., Koyanagi A., Solmi M., Carvalho A.F., Kim E. (2023). Causes for Retraction in the Biomedical Literature: A Systematic Review of Studies of Retraction Notices. J. Korean Med. Sci..

[5] Jefferson T., Alderson P., Wager E., Davidoff F. (2002). Effects of editorial peer review: A systematic review. JAMA.

[6] Okyay R.A., Kocyigit B.F., Qumar A.B., Yessirkepov M., Sumbul H.E. (2025). Fifty Years of Retracted Medical Publications From 1975 to 2024: A Comprehensive Analysis of Trends, Reasons, and Countries Using the Retraction Watch Database. J. Korean Med. Sci..

[7] Rivera H., Teixeira da Silva J.A. (2021). Retractions, Fake Peer Reviews, and Paper Mills. J. Korean Med. Sci..

[8] Schroter S., Black N., Evans S., Godlee F., Osorio L., Smith R. (2008). What errors do peer reviewers detect, and does training improve their ability to detect them?. J. R. Soc. Med..

[9] Shi Q., Wang Z., Zhou Q., Hou R., Gao X., He S., Zhao S., Ma Y., Zhang X., Guan Q. (2021). More consideration is needed for retracted non-Cochrane systematic reviews in medicine: A systematic review. J. Clin. Epidemiol..

[10] Xu C., Fan S., Tian Y., Liu F., Furuya-Kanamori L., Clark J., Zhang C., Li S., Lin L., Chu H. (2025). Investigating the impact of trial retractions on the healthcare evidence ecosystem (VITALITY Study I): Retrospective cohort study. BMJ.

[11] Huber J., Inoua S., Kerschbamer R., Konig-Kersting C., Palan S., Smith V.L. (2022). Nobel and novice: Author prominence affects peer review. Proc. Natl. Acad. Sci. USA.

[12] Scanff A., Naudet F., Cristea I.A., Moher D., Bishop D.V.M., Locher C. (2021). A survey of biomedical journals to detect editorial bias and nepotistic behavior. PLoS Biol..

[13] Horta H., Jung J. (2024). The crisis of peer review: Part of the evolution of science. High. Educ. Q..

[14] Bazoukis G. (2020). Prestige bias--an old, untreated enemy of the peer-review process. Hippokratia.

[15] Mani J., Makarevic J., Juengel E., Ackermann H., Nelson K., Bartsch G., Haferkamp A., Blaheta R.A. (2013). I publish in I edit?--Do editorial board members of urologic journals preferentially publish their own scientific work?. PLoS ONE.

[16] Resnik D.B., Gutierrez-Ford C., Peddada S. (2008). Perceptions of ethical problems with scientific journal peer review: An exploratory study. Sci. Eng. Ethics.

[17] Trinh T.M. (2024). Can training doctoral students to participate in peer review alleviate the shortage of peer reviewers in academic publishing?. Sci. Ed..

[18] Wilhite A.W., Fong E.A. (2012). Scientific publications. Coercive citation in academic publishing. Science.

[19] Bauchner H., Rivara F.P. (2024). Use of artificial intelligence and the future of peer review. Health Aff. Sch..

[20] Gibney E. (2025). AI tools are spotting errors in research papers: Inside a growing movement. Nature.

[21] Alnaimat F., AlSamhori A.R.F., Hamdan O., Seiil B., Qumar A.B. (2025). Perspectives of Artificial Intelligence Use for In-House Ethics Checks of Journal Submissions. J. Korean Med. Sci..

[22] Sun L., Chan A.T., Chang Y.S., Dow S.P. (2024). ReviewFlow: Intelligent Scaffolding to Support Academic Peer Reviewing. IUI ‘24: Proceedings of the 29th International Conference on Intelligent User Interfaces.

[23] Tyser K., Lee J., Shporer A., Udell M., Te’eni D., Drori I. (2023). OpenReviewer: Mitigating Challenges in LLM Reviewing. OpenReview.net.

[24] Using AI in Peer Review and Publishing. https://us.sagepub.com/en-us/nam/using-ai-in-peer-review-and-publishing.

[25] Leung T.I., de Azevedo Cardoso T., Mavragani A., Eysenbach G. (2023). Best Practices for Using AI Tools as an Author, Peer Reviewer, or Editor. J. Med. Internet Res..

[26] Bergstrom C.T., Bak-Coleman J. (2025). AI, peer review and the human activity of science. Nature.

[27] Envisioning a Hybrid Model of Peer Review. https://theeditorialhub.com/scholarly-publishing/envisioning-a-hybrid-model-of-peer-review/.

[28] Eisen M.B., Akhmanova A., Behrens T.E., Harper D.M., Weigel D., Zaidi M. (2020). Implementing a “publish, then review” model of publishing. eLife.

[29] Omar M., Sorin V., Collins J.D., Reich D., Freeman R., Gavin N., Charney A., Stump L., Bragazzi N.L., Nadkarni G.N. (2025). Multi-model assurance analysis showing large language models are highly vulnerable to adversarial hallucination attacks during clinical decision support. Commun. Med..

[30] Majovsky M., Cerny M., Kasal M., Komarc M., Netuka D. (2023). Artificial Intelligence Can Generate Fraudulent but Authentic-Looking Scientific Medical Articles: Pandora’s Box Has Been Opened. J. Med. Internet Res..

